# Description of the use of multicriteria to support pricing and reimbursement decisions by European health technology assessment bodies

**DOI:** 10.1186/s12913-021-06784-8

**Published:** 2021-08-14

**Authors:** David Elvira, Mercè Obach, Caridad Pontes

**Affiliations:** 1 Departament de Farmacologia, de Terapèutica i de Toxicologia, Universitat Autònoma de Barcelona, Sabadell (Barcelona), Spain; 2 Servei Català de la Salut, Gerència del Medicament, Barcelona, Spain

**Keywords:** Health technology assessment, Multicriteria assessment methods, Reimbursement systems

## Abstract

**Background:**

Heterogeneity in drug access throughout Europe may be influenced by differences in drug-assessment strategies. The EUnetHTA’s assessment core model (EUnetHTA-core) and the EVIDEM’s multicriteria framework are reference methodologies in this context, the latter including a wider compromise between non-contextual and contextual criteria. Compliance of 37 European Health Technology Assessment bodies (HTAb) with EUnetHTA-core has been reported, but the use of EVIDEM by this HTAb is still unknown.

**Methods:**

To describe the uptake and use of multicriteria approaches to evaluate drug value by European HTAb using EVIDEM as reference framework, a multicriteria framework was obtained based on EVIDEM model. The criteria used for drug appraisal by HTAb was extracted from the EUnetHTA report, and completed through search of websites, publications and HTAb reports. Use of EVIDEM assessment model in 37 European HTAb has been described semi-quantitatively and summarized using an alignment heatmap.

**Results:**

Aligned, medium or misaligned profiles were seen for 24,3%, 51,4% and 24,3% of HTAb when matching to EVIDEM dimensions and criteria was considered. HTAb with explicit responsibilities in providing specific advice on reimbursement showed more aligned profiles on contextual and non-contextual dimensions.

**Conclusions:**

EUnetHTA’s core model is limited in assessing medicines while EVIDEM’s framework provides contextual dimension used by some HTAb in Europe that can be escalated to other agencies. Most of the 37 European HTAb have room to broaden their contextual assessment tools, especially when social and medical perception of need requires to be explicit to support payer’s decision on reimbursement.

**Supplementary Information:**

The online version contains supplementary material available at 10.1186/s12913-021-06784-8.

## Introduction

One of the major cost drivers in the European healthcare systems is the pharmaceutical ‘innovation’; even considered more relevant than demographics [1]. At the same time, it is also recognized as one of the main contributors to the improvement of the population health status [2].

According to the most recent study from the Organization for Economic Co-operation and Development (OECD) [3], pharmaceutical expenditure accounts for a percentage that range between 11.4% (UK) and 19.1% (Spain) of total healthcare expenditure across the five largest European drug markets (France, Germany, Italy, Spain, and the UK). Specifically, the oncological and hematological drugs are leading the budget impact related to pharmaceutical innovation. The impact is driven by the expansion of multiple new indications normally based on a molecular definition that restricts the population to be treated and the drug ends up being designated as orphan-like medicines [4]. As estimated by a recent study [5, 6], the healthcare expenditures on cancer in the European Union member states represented roughly 6% of total healthcare expenditures. The steady increase of oncology costs is aligned with the disease increasing incidence, the progressive reduction of mortality as well as high prices, in contrast with the less robust evidence data on outcomes [7].

A recent study [5] estimated that 40% of the new orphan drugs authorized in Europe are related to rare neoplastic disorders, and compare to non-oncologic indications, the authorization is received at more advance stages of the clinical development and recognizing a higher potential clinical benefit. From 2009 to 2013, only 35% the 68 oncology indications approved by the European Medicines Agency (EMA) showed a significant prolongation of survival and only 10% showed an improvement in quality of life at the time of market approval. The magnitude of the benefit on overall survival ranged from 1.0 to 5.8 months (median 2.7 months). In the subsequent post marketing period (3,3 years later) there was evidence for extension of life in 7% of the previous authorizations and reported benefit on quality of life in 11% of the cases [8].

Occasionally, when the drug can cover clinical unmet needs with poor prognosis, the regulators trend to accept less and poorer evidence and include especial approvals, such as conditional approval related to further of adequate risk benefit rate in real world, after commercialization, or approval under exceptional circumstances when this may not be achieved, in order to ensure an earlier access to market. As described recently [9] the potential benefit of patients’ early access to new medicines in areas of high unmet medical need, and based on initial data only, have relevant implications in terms of medical and economic costs (opportunity costs of using alternative more efficient treatments available for patients). Several initiatives have been developed in Europe to address these challenges of funding premium priced products related to clear medical unmet needs but with limited evidence [10]. New access management models of these drugs have been promoted across Europe recently, especially for advance therapies, orphan drugs and medicines for cancer, and including innovative access schemes as value-based pricing, conditional reimbursement schemes or risk sharing approaches [11]. Despite the smooth increase of these new access schemes, the number of outcome-based solutions is still very limited being the lack of a systematic and harmonized value assessment methodology one of the main limitations [12].

Beyond the general awareness among healthcare authorities to ensure “value for money”, or the link between price and social or clinical value of the pharmaceutical innovation [13], the reimbursement process and value assessment of drugs is still an open debate in Europe [14]. Several methods have been developed to assess the value of drugs and set meaningful prices affordable to health-care systems [15]. These methods are normally based on the clinical benefits of the drugs and partially on value-based pricing (e.g. cost-effectiveness analysis). However, there is neither a consensus nor a European harmonization related to drug-pricing systems and, based on a comparative international policy analysis, value-based approaches to determine the prices of innovative products are diverse [16]: including the implicit clinical value of the quality-adjusted life-years (QALYS), mainly used in UK, Sweden or Australia, or the value classification based on innovation scales (used in France, Italy, Germany, Austria, Canada or Japan) [17]. Normally new drugs classified as an innovative medicine are reimbursed at a higher price than the current therapeutic alternatives; although the amount, type and methodology to set the premium is normally veiled by the healthcare authorities [7].

In Europe, the European Network for Health Technology Assessment (EUnetHTA) was set up in 2006 and includes all EU Member States to provide strategic guidance and policy orientation on the assessment of health technologies (including drugs), by developing policy papers and discussing areas of potential collaboration. During the last decade the network has focused the efforts on the development of common methodologies, piloting and producing joint early dialogues and Health Technology Assessment (HTA) joint assessment reports, as well as developing and maintaining common tools [18]. One of the most relevant tools developed by the network is the HTA Core Model for Rapid Relative Effectiveness Assessment (REA) [19]. The Model is a methodological framework for the collaborative production and sharing of HTA information that defines the content elements to be considered in an HTA and it enables standardized assessment reporting across Europe. Because of the objective of the framework is to share commonly required elements of information, only information that is considered both important and transferable is collected. The model brings a standardized framework that allows a common comparison of the drivers that lead pricing and reimbursement decisions among different European authorities.

HTA Network approach is focused on technical aspects while methods to support alignment of decisions with the compassionate impetus of healthcare systems is lacking [20]. In many countries, healthcare authorities are including a broader approach to assess the pharmaceutical products (especially in therapeutic areas like oncology and rare diseases) [21]. EVIDEM [22] (Evidence and Value Impact on Decision Making) was developed based on an analysis of the foundations of healthcare systems, the reasoning underlying decisions and fair processes, and has become a reference for multicriteria decision approaches in this setting. It includes the concept of reflective multicriteria assuming decision-makers are guided by a generic interpretative frame rooted in the baseline values of the healthcare systems, drawing on several domains of knowledge including healthcare ethics, evidenced-based medicine, health economics or health technology assessment approaches. A multicriteria analysis provides an effective approach to increase the legitimacy of decisions. Beyond a tool, reflective multicriteria pioneered by EVIDEM is geared to transform the vision of the value of healthcare interventions and how they might contribute to relevant, equitable and sustainable healthcare systems. EVIDEM can be used to compare various healthcare interventions and prioritize its implementation using a performance matrix underpinned in the several dimensions and criteria defined by the framework [20].

EVIDEM criteria overlap with EUnetHTA-core except for 4 non-contextual and 3 social criteria, which are absent or partially included in the EUnetHTA framework. Inversely, 2 EUnetHTA criteria are absent in the EVIDEM framework (Table 1).

**Table 1:** EVIDEM and EUnetHTA criteria correspondence

	EVIDEM CRITERIA	EUnetHTA CRITERIA
**NON-CONTEXTUAL CRITERIA**		
Disease severity	• Effect of disease on life-expectancy • Effect of disease on morbidity (includes disability and function) • Effect of disease on patients’ quality of life • Effect of disease on caregivers’ quality of life	Methodology requirements for the clinical assessment compared to the HTA Core Model for REA - SEVERITY DEFINITION
		A description of the health problem and current use of technology are included in assessments
Size of affected population	• Prevalence • Incidence	Methodology requirements for the clinical assessment compared to the HTA Core Model for REA - POPULATION
		A description of the health problem and current use of technology are included in assessments
Unmet needs	• Unmet needs in efficacy • Unmet needs in safety • Unmet needs in patient reported outcomes • Patient demand	A description of the health problem and current use of technology are included in assessments
		Systematic search strategies applied to evidences (HEALTH PROBLEM - CURRENT TECHNOLOGY USE)
Comparative effectiveness	• Magnitude of health gain • Percentage of the target population expected to achieve the anticipated health gain • Onset and duration of health gain • Sub-criteria for the measure of efficacy specific to the therapeutic area	The comparator is supported by evidence on its efficacy profile for the respective clinical indication/population
		Assessments analyze clinical effectiveness / efficacy (added therapeutic value)
		Systematic search strategies applied to evidences (EFFICACY-EFFECTIVENESS)
Comparative safety/tolerability	• Adverse events • Serious adverse events • Fatal adverse events • Short-term safety • Long-term safety • Tolerability	The comparator is supported by evidence on its safety profile for the respective clinical indication/population
		Assessments analyze safety
		Systematic search strategies applied to evidences (SAFETY)
Comparative patient-perceived health	• Improvement in health-related quality of life • Impact on autonomy • Impact on dignity • Convenience / ease of use / mode & setting of administration	QALYs applied
		Assessments analyze patient aspects
		Assessments include a separate ethical analysis
		Systematic search strategies applied to evidences (PATIENT ASPECTS)
Type of preventive benefit	• Eradication, prevention, reduction in disease transmission, reduction in the prevalence of risk factors). Public health perspective.	Not available
Type of therapeutic benefit	• Symptom relief, prolonging life, cure	Assessments include a description of the health problem and current use of technology
Comparative cost consequences – cost of intervention	• Net cost of intervention • Acquisition cost • Implementation/ maintenance cost	Assessments analyze cost, budget impact or include economic evaluation
Comparative cost consequences – other medical costs	• Impact on primary care expenditures • Impact on hospital care expenditures • Impact on long-term care expenditures	Assessments analyze cost, budget impact or include economic evaluation
Comparative cost consequences – non-medical costs	• Impact on productivity • Financial impact on patients • Financial impact on caregivers • Costs to the wider social care system	Assessments analyze social aspects
Quality of evidence	• Validity (study design, agreement among studies) • Relevance (population, disease stage, outcomes) • Completeness of reporting (uncertainty, conflicting results across studies, limited number of studies) • Type of evidence	Sources of evidence included as relevant clinical evidence for the clinical assessment (1- randomized controlled; 2- Nonrandomized prospective; 3- Other observational; 4- Expert Opinion).
		Methodology requirements for the clinical assessment compared to the HTA Core Model for REA
		Formal tools or algorithms for evidence grading applied
		The GRADE approach in routine use
		Plan for how evidence will be synthesized (e.g. evidence tables, meta-analysis, qualitative synthesis)
		Tables and forms are standardized for evidence synthesis and analysis
		Evidence analysis include surrogate endpoints, composite endpoints, PROs, HRQoL measures, indirect comparisons, meta-analysis, relevant group sub-population, key deficiencies in available data, transferability issues, summary of findings
		Sources of evidence on the technology: A. scientific journal publications, B. grey literature (e.g. published reports), C. unpublished data, D. register data, E. administrative data, F. manufacturer data
		Confidential data from manufacturers accepted
Expert consensus/clinical practice guidelines	Current consensus of experts on what constitutes state-of-the-art practices (guidelines	Not available
**CONTEXTUAL CRITERIA**		
Mandate and scope of the healthcare system	Alignment with healthcare plans/systems	Circumstances where HTA reports are provided
Population priorities and access	• Current priorities of health system (e.g. low socioeconomic status; specific age groups) • Special populations (e.g. ethnicity) • Remote communities • Rare diseases • Specific therapeutic areas	Assessments analyze social aspects
Common goal and specific interests	• Stakeholder pressures • Stakeholders barriers • Conflict of interest	Assessments analyze social aspects
Environmental impact	• Environmental impact of production • Environmental impact of use • Environmental impact of implementation • Environmental impact of production • Environmental impact of use • Environmental impact of implementation	Not available
System capacity and appropriate use of intervention	• Organizational requirements (e.g., process, premises, equipment) • Skill requirements • Legislative requirements • Surveillance requirements • Risk of inappropriate use • Institutional limitations to uptake	Assessments include a separate ethical analysis
		Assessments analyze legal aspects
		Assessments analyze organizational aspects
Political/historical/cultural context	• Political priorities and context • Cultural acceptability • Precedence (congruence with previous and future decisions) • Impact on innovation & research • Impact on partnership & collaboration among healthcare stakeholders	Assessments include a separate ethical analysis

Source: reference [20]. *GRADE* Grading of Recommendations, Assessment, Development and Evaluations, *HTA* Health Technology Assessment, *HRQoL* Health Related Quality of Life, *PROs* Patient Reported Outcomes, *QALY* Quality Adjusted Life Years, *REA* Relative Effectiveness Assessment

**Fig. 1:**
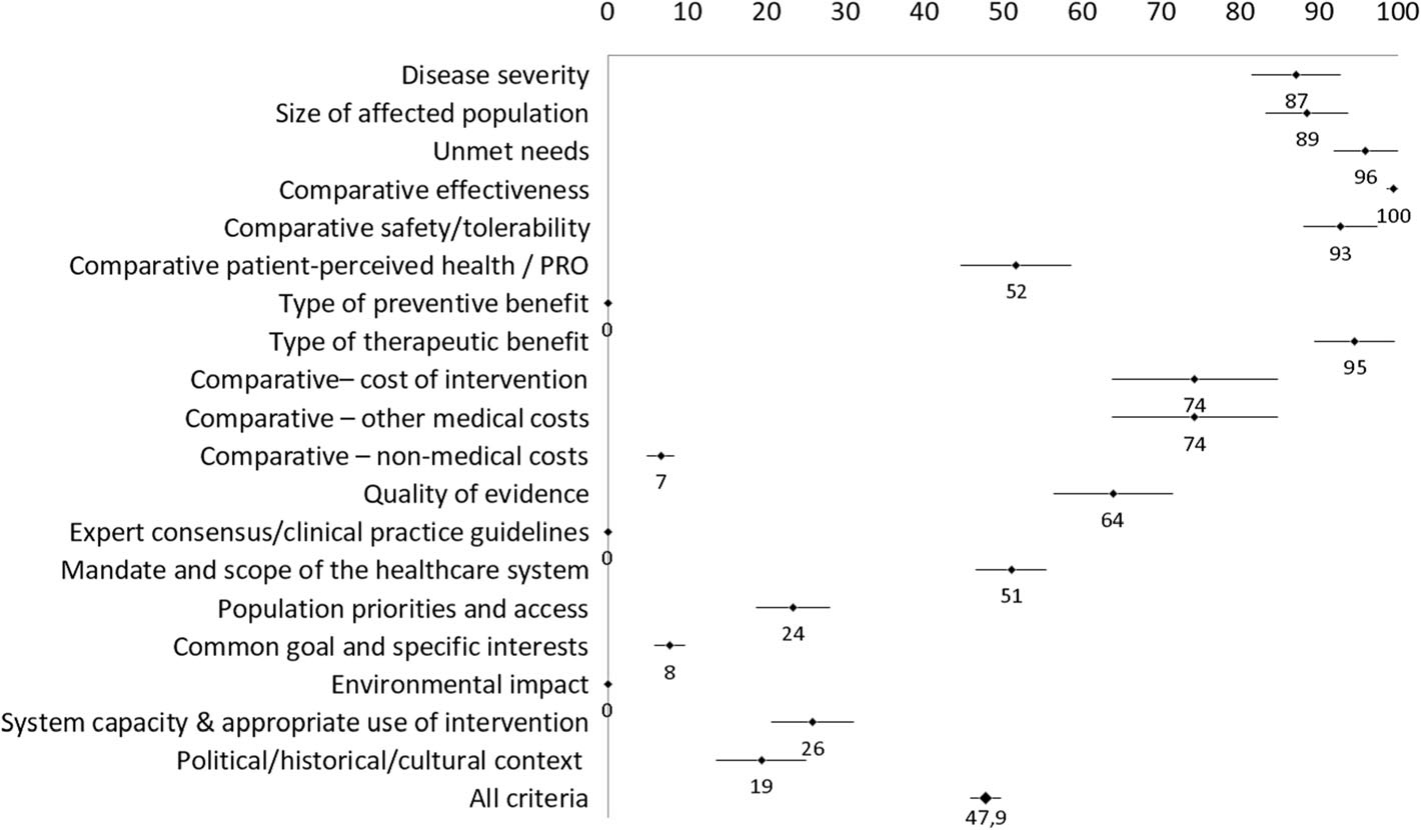
EVIDEM alignment score by dimension

Although multicriteria EVIDEM approach is now applied by several healthcare authorities [23], especially when the social and medical perception of need requires a more holistic assessment framework to support the payer’s decision, a formal and systematic comparison of EUnetHTA’s and EVIDEM’s methodological frameworks and whether European health technology assessment bodies (HTAb) are aligned with the EVIDEM methodology standards is lacking [24]. Since EUnetHTA and EVIDEM frameworks differ in a substantial number of criteria, it is of interest to know the extent of compliance with EVIDEM framework of HTAs as an additional way to explore potential reasons of assessment discrepancies. Despite the compliance of 37 European HTAb with using the supportive criteria for decision making proposed in the EUnetHTA-core framework has been previously reported [18], whether these HTAb do also comply with the wider EVIDEM multicriteria is unknown.

Thus, the main aim of this study is to describe the uptake and use of multicriteria approaches to appraise drug value by 37 European HTAb, using EUnetHTA and EVIDEM as reference frameworks.

## Methods

A quantitative validation of the degree of alignment with the EUnetHTA’s standard framework of 37 European HTAb from 28 countries was done, based on a previous qualitative analysis conducted by the European Commission [18] and an additional thorough search of websites, publications and reports of HTAb. The criteria used for appraisal by the different HTAb were identified and classified, and the matching with the criteria described in the EVIDEM methodological framework were described semi-quantitatively using a heatmap of alignment.

The items reported included those criteria in the HTA Core Model, namely: Relative Effectiveness Assessment (REA) of pharmaceuticals, EUnetHTA methodological guidelines [25] and procedure descriptions [26, 27]. Also, criteria related to the types of technologies assessed, the administrative level (national, regional, institutional) and the formal background (legislation, formal agreement, internal guideline) of certain methodological requirements were also used.

An updated version of EVIDEM framework (v.10) was analyzed in order to assess how the dimensions and criteria included in the EUnetHTA methodological framework fitted within the EVIDEM’s methodological framework.

The EVIDEM framework includes 13 non-contextual dimensions and 6 contextual dimensions (Table 1). The non-contextual dimensions (EVIDEM core-model) include normative aspects combined with the description of the technical knowledge available. Contextual dimensions tailor the framework to the context of decision-making.

**Fig. 2:**
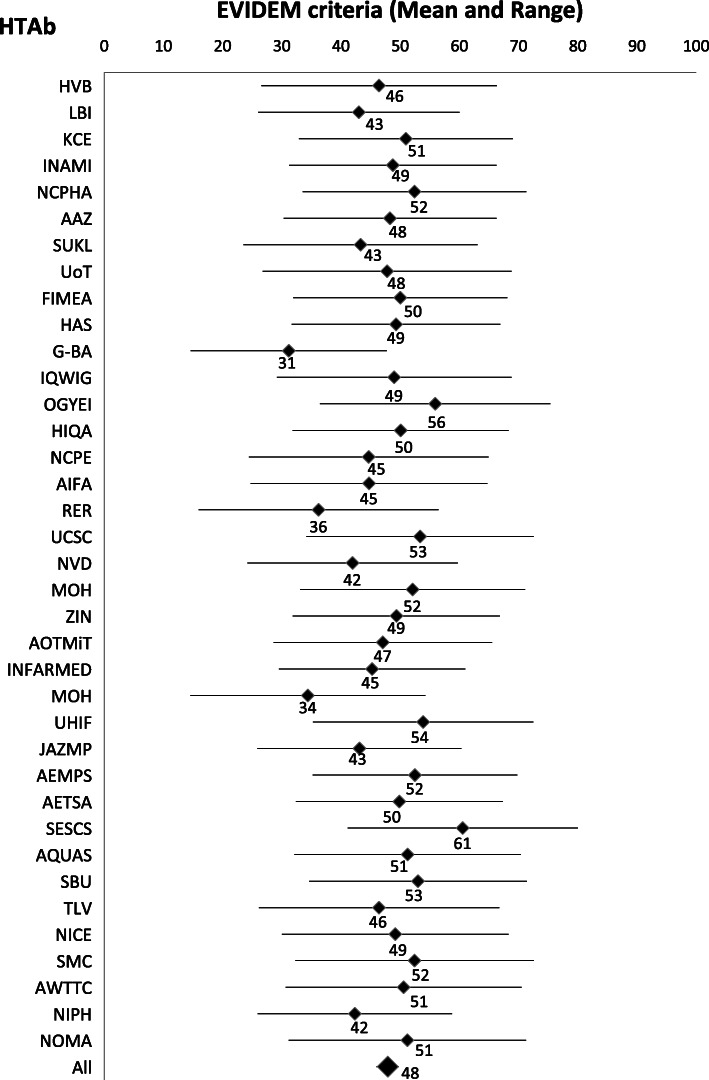
EVIDEM alignment score by HTAb. HTAb: Health Technology Assessment body. SESCS: Servicio de Evaluación del Servicio Canario de Salud; SBU: Swedish Agency for Health Technology Assessment and Assessment of Social Services; HVB: Hauptverband der Österreichischen Sozialversicherungsträger; KCE: Belgian Health Care Knowledge Centre; INAMI-RIZIV: National Institute for Health and Disability Insurance; NCPHA: National Center of Public Health and Analyses; SUKL: State Institute for Drug Control; FIMEA: Finnish Medicines Agency; HAS: Haute Autorité de Santé; IQWiG: Institute for Quality and Efficiency in Health Care; OGYÉI: National Institute of Pharmacy and Nutrition; HIQA: Health Information and Quality Authority; NCPE: National Centre for Pharmacoeconomics; AIFA: Italian Medicines Agency; UCSC: Università Cattolica del Sacro Cuore; ZIN: Zorginstituut Nederland; AOTMiT: Agencja Oceny Technologii Medycznych i Taryfikacji; INFARMED: National Authority of Medicines and Health Products; UHIF: Union Health Insurance Fund; AEMPS: Agencia Española de Medicamentos y Productos Sanitarios; AETSA: Agencia de Evaluación de Tecnologías Sanitarias de Andalucía; AQUAS: Agència de Qualitat i Avaluació Sanitàries de Catalunya; TLV: Dental and Pharmaceutical Benefits Agency; NICE: National Institute for Health and Care Excellence; SMC: Scottish Medicines Consortium; AWTTC: All Wales Therapeutics and Toxicology Centre; NIPH: Norwegian Institute of Public Health; NoMA: Norwegian Medicines Agency; LBI-HTA: Ludwig Boltzmann Institute of Health Technology Assessment; AAZ: Agency for Quality and Accreditation in Health Care and Social Welfare; UoT: University of Tartu; G-BA: Gemeinsamer Bundesausschuss; RER: Regione Emilia-Romagna; NVD: The National Health Service; MOH: Ministry of Health Malta; MOH: Ministry of Health Slovakia; JAZMP: Agency for Medicinal Products and Medical Devices

An HTAb heatmap was developed, where heatmap categories were generated for each EVIDEM’s dimension using as a source the mentioned criteria in the EUnetHTA’s report [18], webs and reports available from the different HTAb analyzed (supplementary file). The contribution (weight) of each mentioned criterion to the final heatmap’s score by dimension was equal and proportioned to the number of criteria by dimension described in Table 1. Only when the mentioned criteria were not fully aligned with the EVIDEM’s criteria, the mention was weighted by 50% of contribution:

$$ Heat\ Score=\left[\left(\sum \# criteria\ mentioned\ by\ dimension\right)/\left(\sum \# total\ criteria\ by\ dimension\right)\right]\ast 100 $$

Descriptive statistics (mean, standard deviation, percentiles) were used to summarize the data and 95% confidence interval for each dimension and HTAb Figs. 1 and 2, and conditional formatting was used to automatically color code each cell using Microsoft Excel (Windows Office 365) so that graded colors were used with green coding for highest alignment (100) and red for lowest alignment (0). Values outside the interquartile range were used to assess alignment with the EVIDEM’s model [28]. HTAbs with and average heat score above the 75th percentile were considered “Aligned” with the EVIDEM model, and those below 25th percentile were considered “Misaligned”. The rest were classified as “Medium” in terms of EVIDEM model’s alignment.

## Results

Most of the non-contextual criteria of EVIDEM are overlapped with the core model of EUnetHTA, except for the type of prevention benefits, non-medical comparative cost consequences, systematic use of expert consensus and use of clinical guidelines to define state-of-the-art, which are not or partially included on the EUnetHTA’s framework (Table 1). Regarding contextual criteria, the assessment of the system capacity and appropriate use of intervention is the most aligned criteria between both frameworks, followed by the political/historical/cultural context assessment, the mandate and scope of the healthcare system, the special population priorities and equity on access criteria. Other social criteria (stakeholders management, conflict of interest assessment or environmental impact assessment) are not reflected in the EUnetHTA’s framework. A systematic general description of the assessed technology and the request of clarification of the assessment process (guidelines and legislation) are key aspects considered by the EUnetHTA analysis that are not explicitly included in the EVIDEM framework.

Most of the non-contextual dimensions (such as disease severity, size of affected population, unmet needs, comparative effectiveness, comparative safety/tolerability or type of therapeutic benefit) show consistently high rates among the HTAb (mean above 85% and standard deviation below 16%); other non-contextual dimensions (type of preventive benefit, comparative non-medical costs, expert consensus) and relevant contextual dimensions (such as population priorities, common goal, environmental impact, system capacity or political/historical/cultural context) are systematically rated low (Table 2).

**Table 2:**
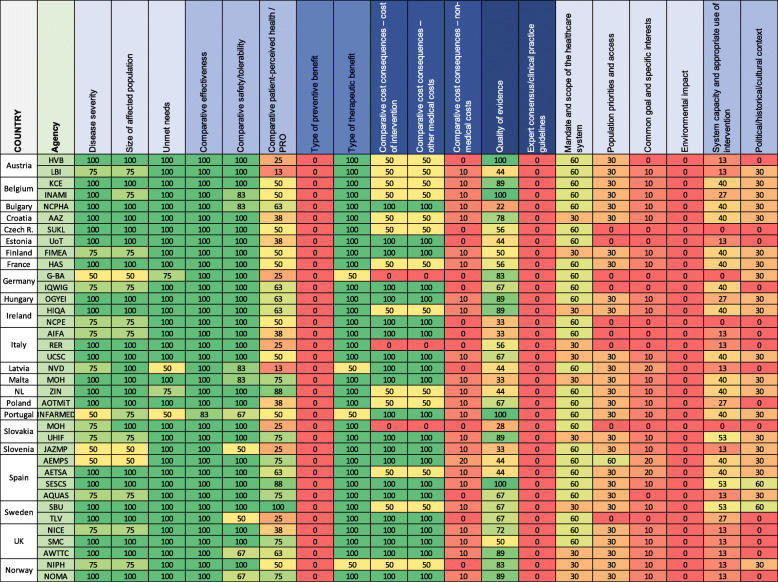
HTAb heatmap of coincidence with EVIDEM framework

*HTA* Health Technology Assessment, *HRQoL* Health Related Quality of Life, *PROs* Patient Reported Outcomes, *QALY* Quality Adjusted Life Years, *REA* Relative Effectiveness Assessment

Color code using Microsoft Excel (Windows Office 365). Graded colors were used with green coding for highest alignment (100) and red for lowest alignment (0)

All HTAb address consistently the health problem and current use of technology, technical characteristics, clinical effectiveness and safety criteria, which are included in the EUnetHTA core model. Choices on comparator, methodology of comparison, endpoints and methods of evidence search and synthesis, are consistently aligned. On the contrary, non-clinical domains, assessment approaches, methodology, modelling algorithms and data are consistently dis-aligned (Table 3).

**Table 3:** EVIDEM heat score by dimension

Criteria	Mean	Standard Deviation	Low 95% CL Mean	Upper 95% CL Mean	25th Percentile	75th Percentile
Disease severity	87.2	17.3	50.0	100.0	75.0	100.0
Size of affected population	88.5	16.2	50.0	100.0	75.0	100.0
Unmet needs	95.9	12.5	50.0	100.0	100.0	100.0
Comparative effectiveness	99.5	2.7	83.3	100.0	100.0	100.0
Comparative safety/tolerability	92.8	14.5	50.0	100.0	100.0	100.0
Comparative patient-perceived health / PRO	51.7	21.9	12.5	100.0	37.5	62.5
Type of preventive benefit	0.0	0.0	0.0	0.0	0.0	0.0
Type of therapeutic benefit	94.6	15.7	50.0	100.0	100.0	100.0
Comparative– cost of intervention	74.3	32.5	0.0	100.0	50.0	100.0
Comparative – other medical costs	74.3	32.5	0.0	100.0	50.0	100.0
Comparative – non-medical costs	6.8	5.3	0.0	20.0	0.0	10.0
Quality of evidence	64.0	23.4	22.2	100.0	44.4	83.3
Expert consensus/clinical practice guidelines	0.0	0.0	0.0	0.0	0.0	0.0
Contextual criteria	0.0	0.0	0.0	0.0	0.0	0.0
Mandate and scope of the healthcare system	51.1	13.9	30.0	60.0	30.0	60.0
Population priorities and access	23.5	14.4	0.0	60.0	30.0	30.0
Common goal and specific interests	7.8	5.8	0.0	20.0	0.0	10.0
Environmental impact	0.0	0.0	0.0	0.0	0.0	0.0
System capacity & appropriate use of intervention	25.9	16.3	0.0	53.3	13.3	40.0
Political/historical/cultural context	19.5	17.6	0.0	60.0	0.0	30.0
GLOBAL	47.9	5.8	31.2	60.5	44.7	51.25

None of the local HTAb had high heat scores with regards to the use of contextual criteria (Table 2). Considering alignment to EVIDEM-driven assessment framework, three patterns of HTAs emerged: “Aligned”, “Medium” and “Misaligned” (Table 4).

**Table 4:** EVIDEM heat score by HTAb

HTAb	Mean	Standard Deviation	Low 95% CL Mean	Upper 95% CL Mean	Degree of Alignment with EVIDEM model
HVB	46.4	44.3	26.5	66.3	Medium
LBI	43.0	37.8	26.0	60.0	Misaligned
KCE	50.9	40.2	32.9	69.0	Medium
INAMI	48.8	39.0	31.2	66.3	Medium
NCPHA	52.4	42.1	33.5	71.3	Aligned
AAZ	48.3	40.0	30.3	66.2	Medium
SUKL	43.3	44.0	23.5	63.0	Misaligned
UoT	47.8	46.8	26.7	68.8	Medium
FIMEA	50.0	40.2	31.9	68.1	Medium
HAS	49.3	39.2	31.6	66.9	Medium
G-BA	31.2	36.8	14.6	47.7	Misaligned
IQWIG	49.0	44.0	29.2	68.8	Medium
OGYEI	55.9	43.3	36.4	75.4	Aligned
HIQA	50.1	40.5	31.9	68.3	Medium
NCPE	44.7	45.0	24.4	64.9	Misaligned
AIFA	44.7	44.5	24.7	64.7	Medium
RER	36.2	45.1	15.9	56.5	Misaligned
UCSC	53.3	42.6	34.2	72.5	Aligned
NVD	41.9	39.5	24.2	59.7	Misaligned
MOH	52.1	42.3	33.1	71.1	Misaligned
ZIN	49.3	38.9	31.9	66.8	Medium
AOTMiT	47.0	41.0	28.6	65.5	Medium
INFARMED	45.3	35.0	29.5	61.0	Medium
MOH	34.4	44.1	14.6	54.2	Aligned
UHIF	53.9	41.4	35.3	72.5	Aligned
JAZMP	43.1	38.3	25.9	60.3	Misaligned
AEMPS	52.5	38.4	35.2	69.7	Aligned
AETSA	49.8	38.9	32.4	67.3	Medium
SESCS	60.5	43.2	41.1	80.0	Aligned
AQUAS	51.3	42.5	32.1	70.4	Medium
SBU	53.0	40.9	34.6	71.4	Aligned
TLV	46.4	45.2	26.1	66.7	Medium
NICE	49.2	42.6	30.0	68.3	Medium
SMC	52.4	44.8	32.3	72.5	Aligned
AWTTC	50.6	44.3	30.7	70.5	Medium
NIPH	42.3	36.5	25.9	58.8	Misaligned
NOMA	51.2	44.6	31.2	71.2	Medium
GLOBAL	47.9	5.8	46.0	49.7	Medium

*HTAb* Health Technology Assessment body, *SESCS* Servicio de Evaluación del Servicio Canario de Salud, *SBU* Swedish Agency for Health Technology Assessment and Assessment of Social Services, *HVB* Hauptverband der Österreichischen Sozialversicherungsträger, *KCE* Belgian Health Care Knowledge Centre, *INAMI-RIZIV* National Institute for Health and Disability Insurance, *NCPHA* National Center of Public Health and Analyses, *SUKL* State Institute for Drug Control, *FIMEA* Finnish Medicines Agency, *HAS* Haute Autorité de Santé, *IQWiG* Institute for Quality and Efficiency in Health Care, *OGYÉI* National Institute of Pharmacy and Nutrition, *HIQA* Health Information and Quality Authority, *NCPE* National Centre for Pharmacoeconomics, *AIFA* Italian Medicines Agency, *UCSC* Università Cattolica del Sacro Cuore, *ZIN* Zorginstituut Nederland, *AOTMiT* Agencja Oceny Technologii Medycznych i Taryfikacji, *INFARMED* National Authority of Medicines and Health Products, *UHIF* Union Health Insurance Fund, *AEMPS* Agencia Española de Medicamentos y Productos Sanitarios, *AETSA* Agencia de Evaluación de Tecnologías Sanitarias de Andalucía, *AQUAS* Agència de Qualitat i Avaluació Sanitàries de Catalunya, *TLV* Dental and Pharmaceutical Benefits Agency, *NICE* National Institute for Health and Care Excellence, *SMC* Scottish Medicines Consortium, *AWTTC* All Wales Therapeutics and Toxicology Centre, *NIPH* Norwegian Institute of Public Health, *NoMA* Norwegian Medicines Agency, *LBI-HTA* Ludwig Boltzmann Institute of Health Technology Assessment, *AAZ* Agency for Quality and Accreditation in Health Care and Social Welfare, *UoT* University of Tartu, *G-BA* Gemeinsamer Bundesausschuss, *RER* Regione Emilia-Romagna, *NVD* The National Health Service, *MOH* Ministry of Health Malta, *MOH* Ministry of Health Slovakia, *JAZMP* Agency for Medicinal Products and Medical Devices

Nine agencies in Bulgary, Hungary, Italy, Malta, Slovakia, Spain, Sweden and UK showed an “Aligned” profile (average heat score above the 75th percentile) with a consistent alignment on non-contextual dimensions and significantly high alignment scores on political/historical/cultural context, system capacity and appropriate use of the intervention.

Most HTAb (19/37; 51%) showed a “Medium” alignment profile. Alignment rates for non-contextual criteria were mainly high (e.g. patient perceived health and quality of evidence dimensions) in these HTAb, and also other contextual dimensions (such as the mandate and scope of the healthcare system, system capacity and appropriate use of the intervention) were rated high. On the contrary, population priorities and access dimension systematically rated below 50%, except for AEMPS.

In 9/37 (24%) HTAb the profile was considered “Misaligned”, with low scores on alignment (average score below 25th percentile) in dimensions such as patients perceived health methods, cost-consequence analysis (cost of intervention and other medical costs) and quality of the evidence. Considering the non-contextual perspective, the German G-BA and the NIPH in Norway show high scores focused and limited to the technical comparison of alternatives (effectiveness, safety and quality of evidence assessment). From the contextual perspective, all the HTAb of this group rated low on the mandate and scope of the healthcare system, population priorities on access, system capacity, appropriate use of the interventions and political/historical/cultural context.

HTAb with explicit responsibilities in providing specific advice on pricing and reimbursement (normally regional agencies in countries with more than one HTAb in place, such as Belgian KCE, German IQWIG, Irish HIQA, Italian UCSC, Portuguese INFARMA, Slovakian UHIF, Spanish SESCS or Swedish SBU) showed higher and similar scores on contextual and non-contextual dimensions.

## Discussion and conclusions

The alignment between EVIDEM and EUnetHTA methodological frameworks is consistently high, especially when assessing domains related to health problem description, current use of the technology, technical characteristics, clinical effectiveness, and safety. However, other non-contextual dimensions of the EVIDEM framework and the EUnetHTA core model are consistently misaligned.

The main EUnetHTA core model criteria, such as clinical effectiveness, safety conditions, health problem description and current use of technology; are consistently addressed by all HTAb. As previously reported [18] the institutions go only partially beyond these criteria and it is normally dependent on the topic of assessment. For those European HTAb directly advising on price and reimbursement decisions, the reported criteria used to support their decisions show a more balanced alignment between both methodological approaches. That conclusion could explain why in many cases, the subnational HTAb in those countries with multiple agencies, are the ones showing a balanced profile among contextual and non-contextual dimensions.

EVIDEM provides a generic interpretive frame (MCDA – Multi-Criteria Decision Analysis – reflective grid) that can be used to elicit individual values and facilitate deliberations through a common structure that includes interpretive scores (quantitative criteria), qualitative impacts (qualitative criteria) as well as narrative comments (all criteria) [21]. EVIDEM framework was designed to minimize the limitations of the deliberation process by ensuring that: generic assessment criteria (either quantitative or qualitative) are included; evidence relevant to each criterion is made available through an efficient synthesis methodology; and face validity is checked at each step of the process (weights, scores and corresponding narratives, aggregated measures). EVIDEM framework is sufficiently flexible to be adapted to the local assessment context, although it also requires consistency in the identification of a set of criteria, scoring scale and weights when assessing a broad range of competing interventions in a specific local context [29, 30].

A holistic approach is required to consistently assess the social and medical needs to support payer’s decision on prices and reimbursement conditions of certain drugs, such as disruptive innovations or orphan drugs, broadening the need of using EVIDEM-like contextual assessment tools by European HTAb.

## Supplementary Information

**Additional file 1.**

## Data Availability

All data generated or analysed during this study are included in this published article and its supplementary information files (supplementary file.xls).

## Abbreviations

EMA: European Medicines Agency
EUnetHTA: European Network for Health Technology Assessment
EVIDEM: Evidence and Value Impact on Decision Making
HTA: Health Technology Assessment
HTAb: Health Technology Assessment Bodies
MCDA: Multi-Criteria Decision Analysis
OECD: Organization for Economic Co-operation and Development
QUALYS: Quality-Adjusted Life-Years
REA: Relative Effectiveness Assessment
